# Titanium Snoreplasty- A New Surgical Technique

**DOI:** 10.22038/ijorl.2019.31930.2051

**Published:** 2020-01

**Authors:** Ahmad Daneshi, Hesam Jahandideh, Farideh Hosseinzadeh

**Affiliations:** 1 *ENT and Head & Neck Research Center, the Five Senses Institute, Iran University of Medical Sciences, Tehran, Iran. *; 2 *Department of Otolaryngology-Head and Neck Surgery, Firoozgar Hospital, Iran University of Medical Sciences, Tehran, Iran. *

**Keywords:** Apnea, Snoring, Titanium, Visual analog scale

## Abstract

**Introduction:**

Based on the previous data, among the general population aged between 30 and 60 years, snoring is observed in 44% and 28% of males and females, respectively. Therefore, it is important to treat snoring to reduce the disruption of the bed partner's sleep and the patients’ own problems. This study aimed to present a minimally invasive procedure which is easy to perform with less tissue damage.

**Materials and Methods:**

This study included 13 patients suffering from primary snoring with soft palate length of 2.5cm or more. All of the patients were examined and their partners were asked to fill-out the relevant questionnaires at baselines, 90 days, 6 months and 1 year after the surgery in order to assess snoring. A crescent strip of oral mucosa along with the underlying muscle were removed under general anesthesia followed by the insertion of a piece of oval-shaped titanium mesh. Moreover, two subjective methods were employed to assess the snoring of all patients.

**Results:**

11 patients were male, and the mean age and the mean body mass index of the patients were 48.69 years and 28.34 kg/m^2^, respectively. The scores obtained from the Visual Analog Scale for snoring loudness before surgery reduced from 7.63 to 3.54, which was statistically significant (P<0.05). None of the patients experienced major complications after surgery; however, there was a partial extrusion of the implant in one case which was managed conservatively with spontaneous healing.

**Conclusions:**

Titanium snoreplasty was successful in the reduction of snoring in this study. This method is a single-stage treatment for simple snoring with the multiple effects of palatal shortening, space increasing, and palatal stiffening.

## Introduction

Snoring occurs when the soft tissues of the upper airway vibrate during sleep (1). The vibration of pharyngeal tissues on inspiration is due to the turbulent airflow through a narrow oropharyngeal or nasopharyngeal space. If the narrowing goes more than snoring, the airflow will be paused due to the upper airway closure at some points which is known as obstructive sleep apnea (OSA) (2).

The American Sleep Disorders Association has classified snoring into mild, moderate, and severe considering the frequency, body position, and disturbance for other people (i.e., spouse, bed partner). Snoring is defined as loud upper airways breathing, without apnea or hypopnea, which is caused by the vibrations of the pharyngeal tissues (3). According to previous data, snoring is observed in the general population aged 30-60 years old and in 44% and 28% of males and females, respectively. Therefore, the treatment of snoring is important since it reduces the disruption of the bed partners' sleep and that of the persons who snore during sleep (1).

There is a risk of hypertension, ischemic heart disease, and cerebrovascular disorders for those who snore. Moreover, some distortion of the rib cage or abdominal wall movements may be observed in polysomnography (PSG); however, simple snoring is not accompanied by arousal, oxygen desaturation, or cardiac arrhythmias (4).

In the absence of OSA, the treatment of snoring is indicated for patients who intend to reduce the disruption of their bed partner's sleep or to relieve themselves from any embarrassment caused by snoring.

Main treatment strategies are conservative approaches (i.e., weight loss, tobacco cessation, reducing or eliminating alcohol consumption, sleeping in the lateral position, and taking measures for maximizing nasal patency), mechanical approaches (i.e., oral appliances, continuous positive airway pressure), and finally surgical approaches, such as uvulopalatopharyngoplasty (UPPP), laser-assisted uvulopalatoplasty (LAUP), radiofrequency assisted uvulopalatoplasty, Pillar implant, palatal implants, injection snoreplasty, cautery-assisted palatal stiffening operation, and uvulopalatal flap which have been described for the treatment of primary palatal snoring (5).The majority of surgical procedures stop soft palate fluttering through the resection of the extra tissues and stiffening of the palate by flaps, since most of the soft palate vibrates in this process. However, the efficacy of these various techniques remains equivocal. Among these methods, one of the newest procedures is Pillar palatal implant system comprising three polyester threads permanently implanted in the soft palate to reduce the airway obstruction in patients with mild to moderate OSA and those who snore during sleep. It is assumed that the soft palate is stiffened due to the direct effects of the implant and surrounding fibrosis. Moreover, it was expected that the soft palate persisted this effect as long as the implants were retained.

Although the available procedures are effective, multiple disadvantages have limited their use in practice. This study aimed to present a new surgical technique called titanium snoreplasty based on the same rationale for Pillar implants; however, it included some unique modifications toward integrating other approaches and maximizing its effectiveness.

## Materials and Methods

All patients with the complaint of snoring underwent PSG. This study included the patients who suffered from primary snoring or mild OSA with soft palate length of 2.5 cm or more (from the uvula base to the junction of the soft and hard palate). Palatal flutter was diagnosed by clinical examination and fiber optic endoscopy of the upper airway during wakefulness (6). On the other hand, the patients with OSA or upper airway resistance syndrome based on PSG results, and those with tonsillar hypertrophy, high tongue position, retrognathia, craniofacial abnormalities, and underlying medical disorders, such as hypertension, bleeding tendency, cardiovascular disorder, and stroke were excluded from the study (7).

Totally, 13 patients were enrolled in this study from March 2014 to 2015. All patients were examined, and they, as well as their bed partners, were asked to complete the questionnaires at baseline, 90 days, 6 months, and 1 year after the surgery to assess snoring. The snoring of all participants was assessed using two subjective methods, namely the visual analog scale (VAS) and snoring scale score (SSS) questionnaires. The VAS was used based on descriptions from the spouses or bed partners to estimate the severity of the patients' snoring using a 10 cm VAS from 0 (no snoring) to 10 (very severe snoring).Additionally, the SSS is a subjective three-item instrument that measures loudness, frequency, and duration of snoring. The SSS is not dependent on age and gender, and it should be completed by the patients' bed partner. Each item consists of four statements measuring the snoring noises. The statement which is closer to describe the patient's snoring noise was selected from each question. Each statement had a predetermined numerical score, which was selected out of the other three options.

 The minimum and maximum scores were 0 and 9 indicating no snoring and very severe snoring, respectively (8) (Table.1). 

A paired 2-tailed student t-test was used to determine if there was a significant difference between the baseline and final scores. The data were analyzed using the IBM® SPSS® software (Version 22.0) through mean and standard deviation. A p-value less than 0.05 was considered statistically significant. It should be noted that written informed consent was obtained from all participants. 

**Table 1:** Snoring Scale Score

Please pick the answer in each of three questions below that best describes your partner’s/spouse’s snoring		
1	How often does your partner/spouse snore?	Score
	A Every night	3
	B Snores on most nights (i.e. more than 50% of nights)	2
	C Snores on some nights (i.e. less than 50% of nights)	1
	D Snores on very rare occasion or never snore	0
2	How much does your partner/spouse snore?	
	A Snores all the time throughout the night	3
	B Snores most of the time throughout the night (i.e. more than 50% of the time)	2
	C Snores some of the time during the night (i.e. less than 50% of the time)	1
	D Hardly snores or no snoring	0
3	How loud is the snore?	
	A Snoring can be heard throughout the floor/flat or louder with the bedroom door closed	3
	B Snoring can be heard in the next room with the bedroom door closed	2
	C Snoring can only be heard in the bedroom	1
	D There is no snoring noise Total score:	0

**Surgical technique**

**Fig 1:**
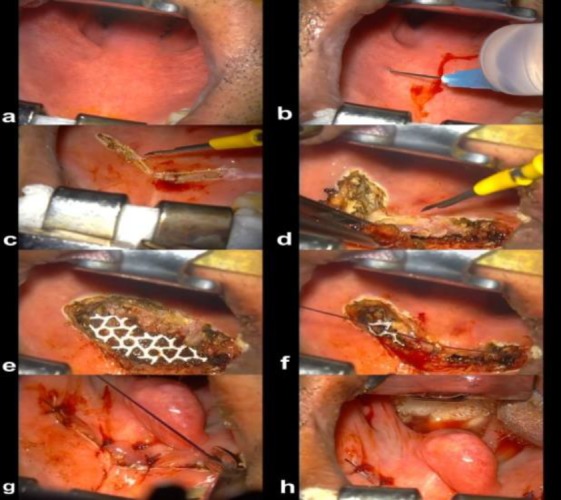
Surgical technique

The surgical procedure was performed under general anesthesia in all patients after fiberoptic evaluation of the palate, while the patients were awake (Fig.1a). 

Subsequently, Lidocaine 2% with adrenaline 1:100 000 was injected locally for local anesthesia facilitating dissection and minimizing bleeding (Fig.1b). 

Afterward, a curved 5 cm incision was made towards the anterior palate 1.5 cm posterior to the junction of the soft and hard palate (Fig.1c). 

In addition, a crescent strip of oral mucosa was removed along with 5 mm of the underlying muscle layer (Fig.1d). 

In the next stage, after proper hemostasis, a piece of flexible, thin, lightweight, and trapezoidal-shape titanium mesh with 2 cm width and 1 cm height was inserted in the surgical site (Fig.1e). The overlying muscle and mucosal layers were repaired in two separate layers using 3/0 Vicryl sutures resulting shortening of the palate (Fig.1f to Fig.1h)

a) Proper patient selection for titanium snoreplasty (having a long soft palate with redundant tissue).

b) Local anesthetic injection.

c) Curved soft palate incision toward the anterior area.

d) Muscle layer removal.

e) Titanium implant in the place.

f) Muscle layer closure.

g) Mucosal layer closure.

h) Final view with shortening and strengthening of the soft palate and increased retropalatal space.

**Fig 2:**
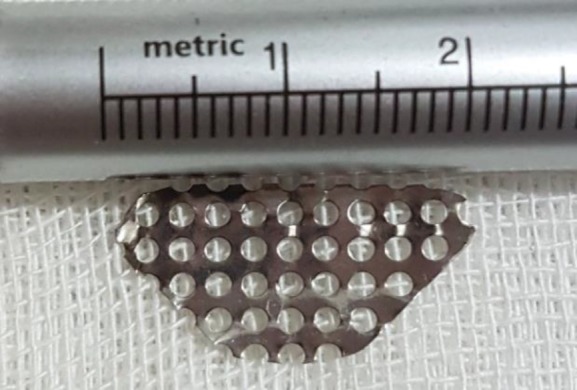
Titanium mesh

**Fig 3:**
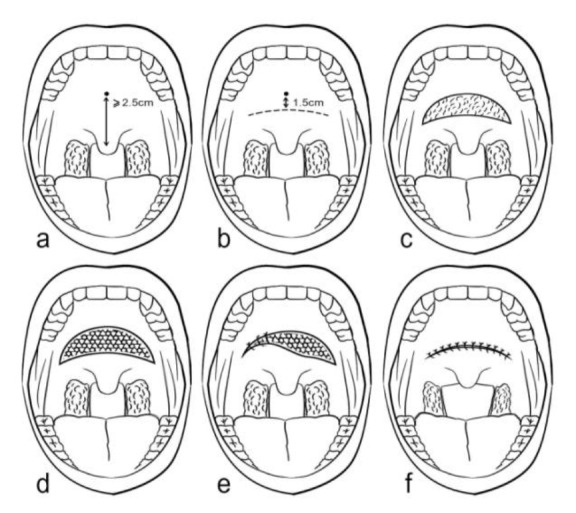
Step-by-step surgical procedure

Detailed specification of titanium implant is shown in Fig.2. The step-by-step surgical procedure is illustrated in Fig.3. Patients were discharged the same day with a 7-day regimen of oral antibiotics and oral rinses three times a day. A liquid and soft diet was prescribed for each patient on the first day, subsequently the regular diet was started on the second day (Fig.3). 

a) Considering the desired length of the soft palate (i.e.,2.5 cm or more).

b) Making a curved incision of 1.5 cm posterior to the junction of the hard and soft palate.

c) Removing a crescent strip of oral mucosa along with the underlying muscle layer.

d) Inserting a piece of titanium mesh.

e) Repairing the overlying muscle and mucosal layers.

f) Shortening and stiffening of the soft palate, intact uvula borders and surfaces, increased space of the pharynx (Final view).

## Results

Out of a total of 13 participants, 11 patients were male. The mean age of the patients was 48.69 ±11.90 years, and the participants' mean body mass index was 28.34 (±3.56) kg/m^2^.

The mean snoring loudness before the surgery using VAS was 7.63±0.63; however, it was reduced to 3.54±0.51 one year after the surgery, which was statistically significant. Meanwhile, the SSS values reduced from 6.84±0.68 preoperatively to 4.38±0.50 one year postoperatively (P<0.05). None of the patients experienced major complications after the surgery; however there was a partial extrusion of the implant in one case, which was managed conservatively with spontaneous healing. All of the patients were discharged on the day of surgery.

## Discussion

Snoring causes many social and lifestyle problems. However, primary snoring with no sleep-disordered breathing is not very harmful to the patient’s health. Therefore, the main purposes of the treatment in this group of patients and mild OSA are the improvement of breathing and reducing snoring (9).

Minimally invasive and less painful procedures are the first choice for both patients and physicians regarding the treatment of snoring (2). While several researches have shown significant improvements, some treatments even worsened the snoring or apnea of patients (9).

The UPPP, LAUP, and radiofrequency ablation decrease snoring by reducing the palate and surrounding tissue volume and/or increasing stiffness via postsurgical scarring. However, significant complications, such as severe pain, difficulty in swallowing, dryness, foreign-body sensation, velopharyngeal insufficiency, nasopharyngeal stenosis, or frequent recurrence of snoring are the main disadvantages of these methods (2).

A new surgical technique was proposed in this study that benefited from multiple previous familiar concepts in snoring treatment. In this technique, both soft palate shortening and stiffening come into effect. Since there is no similar study in the literature, the results should be compared with the findings of the studies which used similar techniques, such as Pillar implants. According to the literature, Pillar implants seem to have a significant effect on snoring and the treatment of mild-to-moderate OSA (2).

The insertion of Pillar implants in the soft palate generates an inflammatory reaction that leads to the formation of a fibrous capsulation around the implants (10). Although the fibrosis induced by surgery initially makes the surrounding tissue rigid, it is usually started to be loosened over time, which could be considered as one of the reasons why snoring can recur after surgery (11). The authors believe that titanium mesh has a similar role in tissue reaction and has a positive effect on reducing palatal vibration because of its weight; moreover, this effect does not disappear with time. In the same line, Choi et al. have shown the impact of the Pillar implant on the reduction of snoring sound in their meta-analysis. The loudness of the snoring sound using VAS was reduced significantly, compared to pre-procedure values (2). In our study, the VAS scores reduced significantly 3 months after the procedure.

Furthermore, there were significant improvements in the patients' snoring scores, which is in concordance with the results of the published data on the success rates of alternative procedures in reducing snoring scores, such as injection snoreplasty (75% to 92%), LAUP (46% to 90%), and radiofrequency technique (77% to 86.6%) (12).

Additionally, postoperative complications after palatal implantation were found to be uncommon in this study. This finding is consistent with the results of previous studies conducted on palatal implants (13).The patients had no voice changes and foreign body sensation due to the presence of a thin fibrosis tissue during the follow-ups.

 It is worth mentioning that the patients reported no significant complications, such as severe pain, difficulty in swallowing, dryness, velopharyngeal insufficiency, and nasopharyngeal stenosis in this study.

This study has some considerable limitations, including the small number of patients and the bias made by documentation of the loudness of snoring using a subjective scale (i.e., VAS) as reported by the bed partners. Since the VAS is mainly a subjective scale, it was probably being influenced much by a placebo effect. Furthermore, the patients and their family members typically might tend to exaggerate the surgical effect (14). 

Another important limitation was a 1-year- follow-up period after titanium mesh insertion. Therefore, further studies are recommended to conduct more follow-ups for more precise conclusions in this regard. 

## Conclusion

A novel surgical technique used for relieving snoring was proposed in this study, named titanium snoreplasty. The procedure was promising in reducing snoring in the study follow-up period. Titanium snoreplasty is a single-stage treatment for simple snoring which acts by different means. It shortens the palate, increases the space, and also stiffens the palate. In this procedure, the anatomic borders of the uvula and soft palate remain intact and there is no gross anatomic change in the patient’s view. This method minimizes the risk of nasopharyngeal stenosis and regurgitation; moreover, this procedure is not technically challenging and can achieve patient satisfaction with minimal risk and high success rate.
